# Influence of subcutaneous specific immunotherapy on drug costs in children suffering from allergic asthma

**DOI:** 10.1186/2045-7022-3-30

**Published:** 2013-09-03

**Authors:** Thomas Reinhold, Julia Ostermann, Susanne Thum-Oltmer, Bernd Brüggenjürgen

**Affiliations:** 1 Institute for Social Medicine, Epidemiology and Health Economics, Charité, University Medical Center, Berlin, Germany; 2 Allergopharma GmbH & Co. KG, Reinbek, Germany; 3 Institute for Health Economics, Steinbeis University Berlin, Berlin, Germany

**Keywords:** Asthma, Cost-effectiveness analysis, Subcutaneous specific immunotherapy, High-dose hypoallergenic mite preparation

## Abstract

**Background:**

Subcutaneous specific immunotherapy (SCIT) is an effective treatment attenuating the progression of allergic asthma. To date, there is a lack of studies investigating the economic consequences of SCIT on health care expenditures.

**Methods:**

A health-economic piggy-back analysis of SCIT was conducted based on a RCT that enrolled 65 children and adolescents with allergic asthma. Patients were allocated into two groups: A group receiving SCIT with a high-dose hypoallergenic house dust mite preparation plus asthma medication and a control group receiving only asthma medication. For both groups asthma control was achieved before the start of the SCIT treatment and was maintained during the study. Both, costs and cost-effectiveness of SCIT with the high-dose hypoallergenic house dust mite preparation were investigated based on total medication costs, incremental medication costs and treatment effects (measured as lung function), respectively. A bootstrap analysis was performed to validate the results.

**Results:**

A steady decline in medication costs could be observed in the SCIT group one year after treatment start compared to the control group. This cost trend became statistically significant 3 years after SCIT started. The calculated potential savings in the SCIT group correlated with an improved lung function. The distribution of the bootstrap results revealed that the probability of SCIT having a superior effectiveness compared to the control group is around 90%.

**Conclusion:**

SCIT with a high-dose hypoallergenic preparation received by children and adolescents suffering from mite induced allergic asthma reduces the allergic medication intake and has cost-saving effects. Additional costs associated with SCIT may be completely compensated by drug cost savings 4 years after end of SCIT. Additionally, SCIT is superior compared to routine care as measured by the lung function that improved in SCIT-treated patients. Trial registration: (EudraCT no. 2004 – 003892 – 35).

## Background

Asthma is one of the most frequent chronic diseases, with about 300 million patients being affected worldwide [1]. In children, asthma is even the most frequent chronic disease [2]. Considering the age-specific prevalence of asthma, it becomes apparent that the asthma prevalence peaks in early childhood and then declines steadily. The German KIGGS study reported the lifetime prevalence of asthma in children and adolescents to be 4.7% [3]. However, this number might overestimate the prevalence, as the answers were derived from self-responses. Quality of life is reduced in children and adolescents suffering from asthma [4]. The patients often have a limited ability to participate in physical activities and are not able to sleep through the night [5]. Asthma therefore poses a significant burden of disease for the affected patients. The annual mean sick leave days in employed asthma patients are around 23 days [6]. In Germany the total direct costs of asthma were measured to be about 2.35 billion euro; the costs for sick leave days caused by asthma were estimated to be 242 million euro for the year 2006 [7]. This demonstrates that asthma is an important economic factor for the health system.

Unlike common antiallergic drugs specific immunotherapy (SIT) is the only treatment for allergic patients that treats the cause of the disease [8]. A specific type of this therapy is the subcutaneous immunotherapy (SCIT). Here, allergen extracts are injected subcutaneously [9]. Allergen-specific subcutaneous immunotherapy (SCIT) is a well-established treatment for mild to moderate asthma. A Cochrane review demonstrated that SCIT is an effective treatment for reducing asthma symptoms as well as medication use [10]. With the decrease in medication use, we hypothesized that the costs in SCIT-treated children should decrease as well.

To our knowledge the cost-effectiveness of SCIT to date in Germany has primarily been demonstrated in model-based approaches [11,12], as opposed to actual patient data. This paper therefore aims to analyse the economic consequences on medication use and the cost-effectiveness of SCIT in children and adolescents with asthma using data from a randomized controlled trial.

## Methods

### Study design

The present health economic piggy back analysis was conducted based on a randomized, controlled multicenter study (EudraCT-Nr. 2004 – 003892 – 35) including children and adolescents suffering from allergic asthma (GINA levels II and III). Before randomization patients’ minimal requirement for the inhaled corticosteroid (ICS) dose to achieve asthma control was determined in the baseline phase from September 2005 to February 2006 which represents the time of the highest exposure to house dust mites. Data from patient diaries during the baseline year build a sound and reliable basis of an individual patient status prior to the start of treatment. After achieving asthma control, patients were randomized into an intervention group, receiving a subcutaneous specific immunotherapy with house dust mite allergoid Acaroid® in addition to standard asthma medication, or into a control group with standard asthma medication alone. The patients were compared during a mean follow up period of 3 years. The study including the present health economic analysis, was approved by an ethics committee in accordance to the ethical principles that have their origin in the Declaration of Helsinki, and that are consistent with GCP and the applicable regulatory requirements.

The primary endpoint of the study was the change in the ICS dose steps required to achieve asthma control (according to GINA [13]) in children treated for two years with SCIT compared with children on ICS alone. The ICS dosages in both groups during the third treatment year were described elsewhere [14]. For details on inclusion and exclusion criteria and detailed statistical methods see the publication of Zielen et al. 2010 [15].

### Economic assessment and outcomes

The economic evaluation was performed using a longitudinal drug cost-analysis, a break even analysis and a cost-effectiveness analysis.

During the study, patients were asked to keep a diary about their total medication use (allergic medication as well as other drugs) and to give information whether a medication intake was associated with their allergic suffering or whether it was related to other non-allergic diseases. The drug consumption was monetarily valued in two ways : If it was applicable to extract the exact quantity of drug intake from patients diaries, the monetarily valuation was calculated using quantity-based prices (e.g. price per milligram) of the German Rote Liste 2011 [16]. If patients documentation on the quantity of the active ingredients was insufficient, we used official DDD-prices (defined daily dose) provided annually by the German Drug-Prescription report [17]. Unfortunately, further resource consumption, such as outpatient stays or hospitalizations, could not be included in the analysis. Next, the costs for SCIT with Acaroid® were considered, and an expected break even-point was calculated. Therefore, we assumed that potential cost savings, realizable for SCIT patients in the third treatment year, would be stable over time after the study. Additionally, we decided to discount the modelled future savings after the third treatment year using a discount rate of 3% to consider a longer time horizon. The underlying SCIT costs per patient treated with Acaroid® in 2012 were assumed to be about 1,597 euro over the 3-years intervention period (perennial therapy, maintenance therapy every 6 weeks, net prices including 19% value added tax, 16% manufacturer sales discount and a price level of August 1, 2009 due to legal price stop).

The effectiveness measure for the following health economic cost-effectiveness analysis was the lung function, expressed as the mean annual morning peak flow (in l/min) after SCIT onset. For cost-effectiveness analysis we hence calculated the mean annual morning peak flow during the 3 years after SCIT began in relation to the mean annual total costs (including SCIT costs) associated with treatment arms. To get a measure of uncertainty we used non-parametric bootstrapping [18]. Therefore, the original sample was bootstrapped 1,000 times to obtain 1,000 means for cost and effect differences that were subsequently plotted in a cost-effectiveness plane.

### Statistical analysis

Socioeconomic data at baseline were analysed using Student’s t-test for comparing continuous variables and Chi-Square test for dichotomous variables. Drug costs in both groups were analysed using Mann–Whitney-U test. For cost-effectiveness analysis, an analysis of covariance (ANCOVA) was conducted to adjust all values of costs and effects for age and the respective peak flow baseline value. The significance level was defined to be 5% (two-sided). For inferential statistics, we used SPSS© version 20. Finally, we used MS Excel© 2007 to model bootstrapped cost-effectiveness analyses.

## Results

### Baseline characteristics

**Figure 1:**
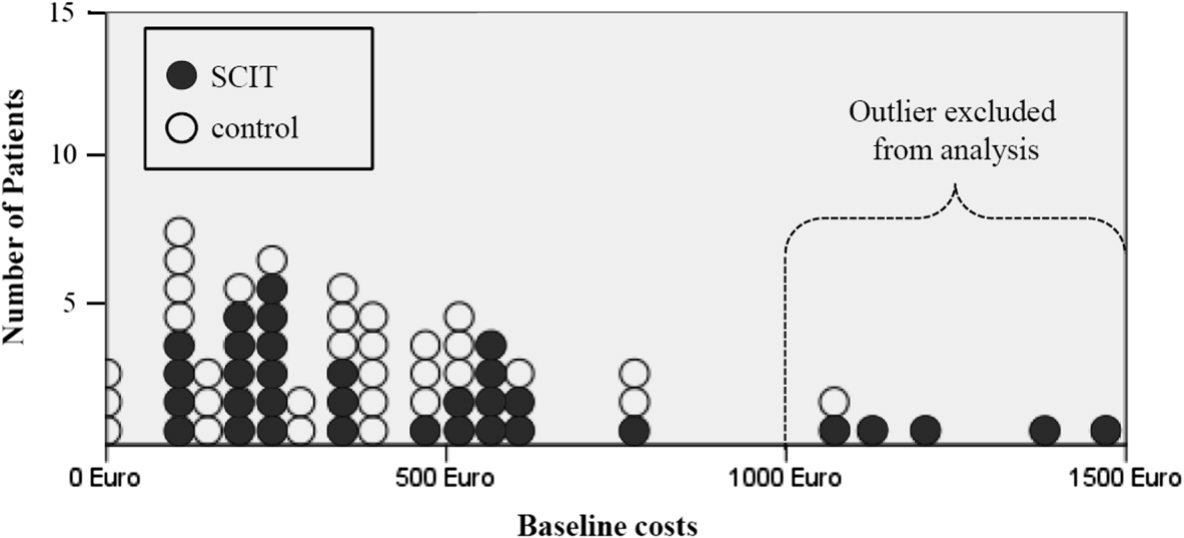
Total baseline drug costs per year and outlier analysis (each dot represents one patient).

A total of 65 patients were initially recruited and randomized into two groups (33 SCIT, 32 controls). The mean age of these patients was 10.0±SD 3.1 years in the SCIT group and 10.6±SD 2, 9 years in the control group. In both groups, about 30% of patients were female. We found no significant differences in the average severity of asthma (GINA) or the peak flow measurements before the SCIT. Differences, however, were detectable with regard to the allergic-drug costs (434 euro per year for SCIT, 296 euro per year in controls, p = 0.130) as well as the resulting total drug costs before the SCIT intervention started (485 euro per year for SCIT, 345 euro per year in controls, p = 0.083). These differences were due to the fact that before the intervention started a total of 6 patients (5 SCIT, 1 control) were identified as outliers with calculated annual total drug costs of more than 1,000 euro per year. These patients were excluded from the following analyses (see Figure  1). The important baseline characteristics before and after outlier exclusion are presented more detailed in Table  1.

**Table 1:** Baseline characteristics of study participants

**Item**	**SCIT**	**Control**	**p-value**
*Baseline characteristics including outliers (all randomized patients)*			
n	33	32	
Female proportion [n (%)]	11 (33.3%)	10 (31.2%)	0.534
Mean age in years [mean (SD)]	10.0 (3.1)	10.6 (2.9)	0.403
GINA level 2 [n (%)]	26 (78.8%)	26 (81.2%)	0.525
GINA level 3 [n (%)]	7 (21.2%)	6 (18.8%)	
Mean peak flow in l/min [mean (SD)]	296 (101)	315 (91)	0.444
Mean annual costs for allergic-drugs before intervention onset in euro [mean (SD)]	434 (332)	296 (220)	0.130
Mean annual costs for non-allergic-drugs before intervention onset in euro [mean (SD)]	51 (87)	50 (98)	0.571
Mean total annual drug costs before intervention onset in euro [mean (SD)]	485 (377)	345 (245)	0.083
*Baseline characteristics without outliers (all analysed patients)*			
n	28	31	
Female proportion [n (%)]	8 (28.6%)	10 (32.3%)	0.785
Mean age in years [mean (SD)]	10.4 (3.2)	10.8 (2.8)	0.628
GINA level 2 [n (%)]	21 (75.0%)	25 (80.6%)	0.417
GINA level 3 [n (%)]	7 (25.0%)	6 (19.4%)	
Mean asthma level GINA [mean (SD)]	2.25 (0.441)	2.19 (0.402)	0.755
Mean peak flow in l/min [mean (SD)]	308 (105)	317 (91)	0.727
Mean annual costs for allergic-drugs before intervention onset in euro [mean (SD)]	315 (180)	273 (183)	0.443
Mean annual costs for non-allergic-drugs before intervention onset in euro [mean (SD)]	33 (54)	48 (99)	0.974
Mean total annual drug costs before intervention onset in euro [mean (SD)]	349 (192)	322 (209)	0.627

### Drug costs analysis

**Figure 2:**
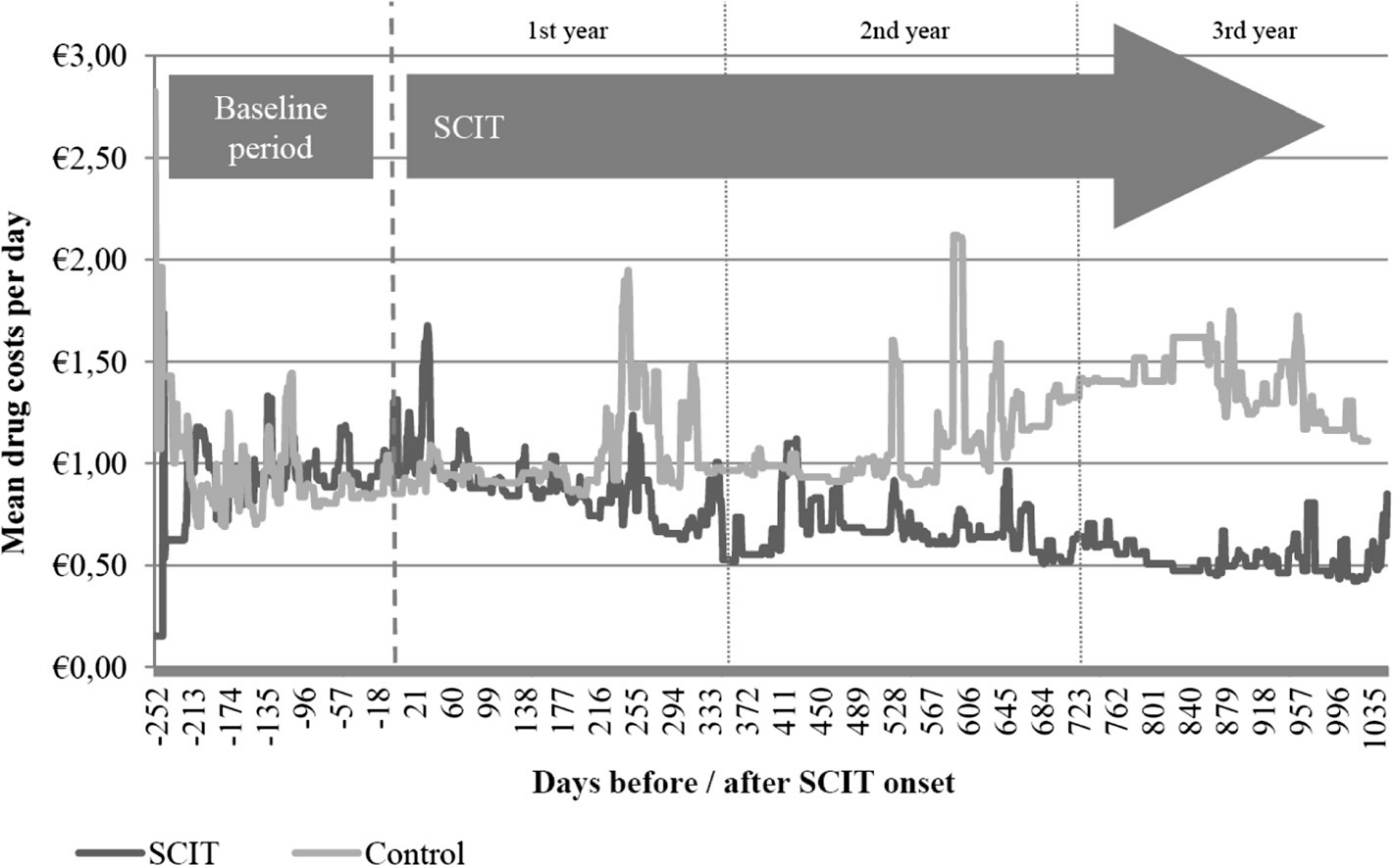
Course of total drug costs during the study on daily basis (descriptive) – not involving SCIT intervention costs.

The descriptive longitudinal total drug cost analysis (see Figure  2) shows a comparable cost trend in the first year after SCIT was started. During the further course of time an increasing cost difference trend was observable favouring patients in the SCIT group. The total drug costs (see Table  2) reach a significant difference in year 3 after the intervention began (193 euro 95% CI [114 to 273] for SCIT, 498 euro 95% CI [293 to 702] for controls, p=0.001). This decreasing course of total drug costs for SCIT-patients was mainly driven by a decrease in costs of allergic medication intake. A significant group difference was already reached after the second treatment year. Expectedly, no intervention effect was detectable with regard to non-allergic drug costs.

**Table 2:** Results on total and allergic/non-allergic drug costs by year after SCIT onset (analytic) – not involving SCIT intervention costs

**Years after SCIT onset**	**SCIT**	**Control**	**p-value**
*Total drug costs in euro [mean (95% CI)]*			
1. year	319 (236 to 402)	364 (262 to 466)	0.716
2. year	250 (168 to 333)	389 (272 to 506)	0.077
3. year	193 (114 to 273)	498 (293 to 702)	0.001
*Allergic drug costs in euro [mean (95% CI)]*			
1. year	270 (197 to 343)	306 (219 to 393)	0.716
2. year	206 (135 to 277)	341 (232 to 451)	0.021
3. year	168 (94 to 242)	453 (249 to 656)	0.002
*Non-Allergic drug costs in euro [mean (95% CI)]*			
1. year	49 (24 to 75)	58 (16 to 100)	0.233
2. year	44 (13 to 75)	47 (7 to 88)	0.262
3. year	25 (2 to 48)	45 (0 to 92)	0.373

### Break even calculation

**Figure 3:**
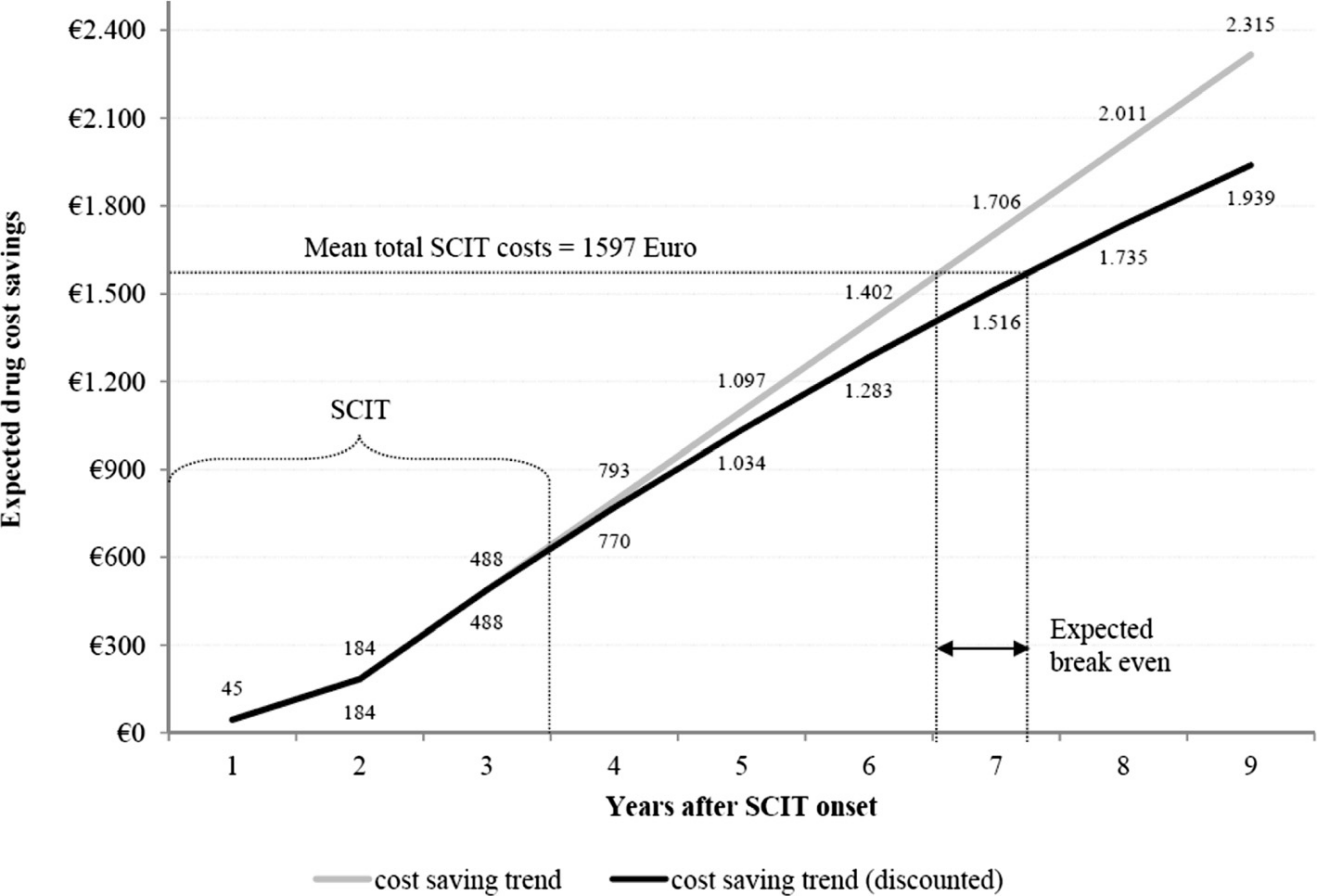
Break-even calculation.

The results of the drug cost analysis indicate cost savings for patients treated with Acaroid®. If these potential drug cost-savings will be extrapolated for a longer time horizon, as it was described in the methods section, the additional costs of 1,597 euro over the 3-years intervention period that are necessary for realizing SCIT, will be expected to be compensated by the drug costs savings about 7 years after SCIT onset or 4 years after the end of triannual SCIT (see Figure  3).

### Cost-effectiveness analysis

**Figure 4:**
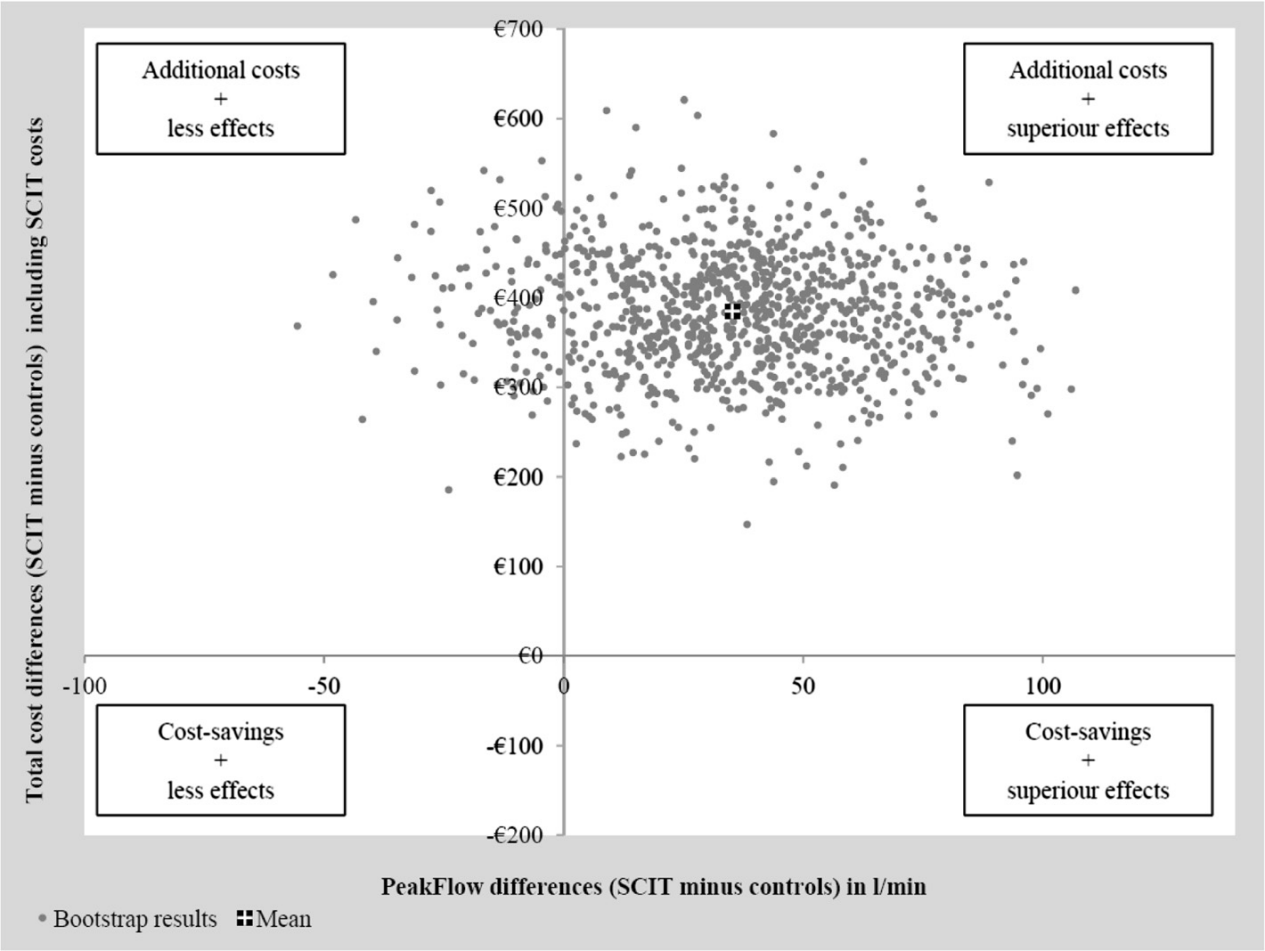
Cost-effectiveness results including SCIT costs.

For cost-effectiveness measurement, the SCIT costs were additionally included and the resulting total costs were evaluated in relation to the observable treatment effects. After consideration of SCIT costs, the adjusted total mean costs per patient over the 3 years treatment duration differed significantly between the groups (770 euro 95% CI [701 to 839] for SCIT, 383 euro 95% CI [317 to 449] for controls, p<0.001). On the other hand, the use of SCIT with Acaroid® seems to be associated with superior effectiveness, measured by changes in peak flow results. The mean annual adjusted morning peak flow over the 3 years of SCIT intervention shows higher values for patients receiving SCIT (369 l/min 95% CI [354 to 385] for SCIT, 334 l/min 95% CI [319 to 348] for controls, p=0.001). The bootstrapped cost-effectiveness results are shown in Figure  4. Most of the dots (900 of 1,000) are located in the upper right hand quadrant of the cost-effectiveness plane, indicating that SCIT is associated with additional costs over the 3-years treatment period, but also with better effectiveness. The probability, that SCIT leads to superior effectiveness compared to controls can be directly derived from the cost-effectiveness plane and is about 90%.

## Discussion

The present investigation indicates SCIT with Acaroid® as a treatment option for children and adolescents suffering from allergic asthma. Thus, allergic medication intake and related drug costs can be reduced, and asthma symptoms improved. After SCIT onset, a cost reduction trend was observable, showing that allergic medication costs decrease from year to year. With regard to SCIT intervention costs, the present analysis indicates, that these costs will be completely compensated by drug cost savings about 7 years after SCIT began resp. 4 years after the end of triannual SCIT.

An important strength of the present analysis is its embedding in a randomized controlled trial, thus reducing the risk for selection bias and increasing the internal validity [19]. As yet, there is a lack of comparable studies on allergic treatment strategies that focus on economic consequences especially in children. A study, published by us in 2008, investigated the economic effects of SCIT using a Markov-model-based approach in age-stratified patients suffering from allergic rhinitis and allergic asthma, and reached similar conclusions [12]. In that study, we calculated a possible break even 10 years post intervention from a society’s perspective.

An important point of discussion in the present analysis is the coverage of costs that could be included for economic assessment. Since the health economic analysis was not initially considered in the planning of the study design, we were not able to get retrospective information on resource consumption outside the medication use. It could be that besides the drug costs savings we detected in the present analysis, further costs will be caused in further health care areas. On the other hand, it seems also plausible, that a reduced need for allergic medication will have a positive effect on other medical claims. To get clearness on other cost effects, it would be desirable to anchorage health economic questions during the planning phase of future studies.

In the present study asthma in all children had to be controlled and the study preparation (Acaroid®) helped to maintain this asthma control. Therefore no emergency visits were observed during the study. Consistently very poorly controlled asthma increases the risk for future severe asthma exacerbations [20,21] and it is well described in literature that patients with uncontrolled asthma have a higher risk for emergency visits [22-24]. Emergency visits are associated with higher total and asthma-related health care costs compared to patients without exacerbations [25]. Our calculation therefore might be considered as very conservative taking only medication costs into account.

Our bootstrapped cost effectiveness results showed that a mean increase of 35 L/min in morning peak flow ( 10% of total peak flow) in SCIT-treated children can be achieved for additional annual costs of about 385 euro. This better peak flow is combined with a fluticasone propionate reduction for asthma control. This is of importance because the use of inhaled corticosteroids in children is often seen critical. In a recent study by Kelly et al. [26] it was shown that the corticosteroid-induced growth retardation in prepubertal children persisted in adulthood although it was not progressive or cumulative. The anti-inflammatory effect of the allergoid preparation demonstrated by an increased lung function is comparable to the effect of inhaled corticosteroids used for asthma control [27].

For break even calculation we assumed the cost savings observed in year 3 to be stable over the following years. This could be criticized as the limited study duration of 3 years did not allow proving this assumption. Yet, a longer treatment effect is not unlikely. For instance, a systematic review published in 2011 found some evidence for beneficial long-term effects of SCIT in allergic children after SCIT termination [28]. Due to the adjusting for differential timing of costs, using a mean annual discounting-rate of 3% after the third year, the future cost savings were valued lower than the present. Although the process of discounting is basically accepted in health economic research [29] it is consistently a subject of discussion, particularly for long-term health care programs where benefits mainly appear in the future [28].

Another limitation could arise from the way we measured the SCIT’s cost-effectiveness. This calculation focused only on the 3 years study duration. During that time, the SCIT took place and caused additional intervention costs while future potential cost savings after year 3 were ignored. Considering a longer time horizon would lead to less additional costs for SCIT and would improve the intervention’s cost-effectiveness.

## Conclusion

SCIT with a hypoallergenic high-dose mite preparation received by children and adolescents suffering from mite-allergic asthma reduces the anti-allergic medication intake of ICS and has cost-saving effects. Additional costs associated with SCIT may be completely compensated by drug cost savings 4 years after the end of triannual SCIT. Additional SCIT is superior compared to only routine care and leads to an improved lung function while asthma control is maintained.

## Competing interests

The study was financially supported by ALLERGOPHARMA GmbH & Co. KG, Germany. The design and implementation of the analysis was solely the responsibility of the authors.

## Authors’ contributions

TR: substantial contributions to conception and design of, or acquisition of data or analysis and interpretation of data, drafting the article or revising it critically for important intellectual content. JKO: drafting the article and revising it critically for important intellectual content. STO: substantial contributions to the discussion, revising the manuscript critically for important intellectual content. BB: final approval of the version to be published. All authors read and approved the final manuscript.
